# Osteochondroma of the Scapula: A Case Report and Literature Review

**DOI:** 10.7759/cureus.30558

**Published:** 2022-10-21

**Authors:** Nouf A Altwaijri, Jameel Fakeeha, Ibrahim Alshugair

**Affiliations:** 1 Orthopedic Surgery, King Saud Medical City, Riyadh, SAU

**Keywords:** scapula, osteochondroma, exostosis, scapular exostosis, scapular osteochondroma

## Abstract

Osteochondromas are bone lesions composed of medullary and cartilaginous bone covered by a cap of hyaline cartilage. The presence of medullary and cortical bone with the continuity of the tumor is pathognomonic for osteochondroma and aid in establishing the diagnosis.

We report a case of a two-year-old girl who presented to our clinic following her mother noticing a palpable, growing, and painful mass on her left scapula. There was no limitation in the range of motion. A clear-cut mass was seen on the dorsal aspect and palpated measuring around 2.5x3 cm. Surgical excision of the mass followed by histologic examination confirmed osteochondroma. Upon follow-up, the patient had no pain and had a full range of left shoulder motion without discomfort or pain.

In conclusion, scapular exostoses are very rare and more so when they present dorsally. Symptomatic lesions can be managed effectively with surgical excision of exostosis.

## Introduction

Osteochondromas are bone lesions composed of medullary and cartilaginous bone covered by a cap of hyaline cartilage. The presence of medullary and cortical bone with the continuity of the tumor is pathognomonic for osteochondroma and aid in establishing the diagnosis [1-4]. They are more of developmental lesions, referred to as exostosis, rather than true neoplasms. Osteochondromas emerge due to a separation of a part of the cartilage of the epiphyseal growth plate, which as a result, herniates through the periosteal bone cuff surrounding the growth plate [2,5-8]. There are multiple differential diagnoses that can be considered, such as Nora's lesion, parosteal osteosarcoma, Dupuytren's exostosis, turret exostosis, subperiosteal hematoma, or juxtacortical chondroma; however, none have medullary continuity [1-4,9-13]. Furthermore, osteochondroma is the most common primary bone tumor comprising around 40% of all benign tumors. Some of the common sites for osteochondroma are the proximal humerus, proximal tibia, and distal femur [14,15]. It was also reported to occur following local radiation therapy [16-23] and total body irradiation in children [16,24,25-35]. Osteochondroma rarely occurs in the scapula; but interestingly, it is the most common tumor occurring in the scapula [36-42], accounting for 4.6% of all bone tumors [38,43]. Osteochondromas are usually discovered incidentally due to their asymptomatic nature [3,44,45]. However, symptoms may present with mechanical compression of adjacent structures, fractures, formation of a bursa or osseous deformities, or even malignant transformation [3,4,45,46]. Malignant transformation is rare and commonly associated with hereditary exostosis [45]. Most reported cases in the literature present an anterior scapular location [47-57] and there are very few reports of posterior surface presentation, as we have reported in this paper [14,42,43,58-61], which further explains why our presented case is unique. Very little information is available regarding dorsal scapular osteochondromas. Furthermore, all reported cases have opted for excision, which was sufficient to alleviate the symptoms [14,42,43,47,48,50-60,62]. Surgical management is usually indicated with the presence of pain, need for cosmesis, complications, high risks of malignant transformation, or uncertain diagnosis [44,45,63-66]. We present a case of a two-year-old girl with painful dorsal scapular osteochondroma, which is extremely rare in the literature in her age group.

## Case presentation

**Figure 1:**
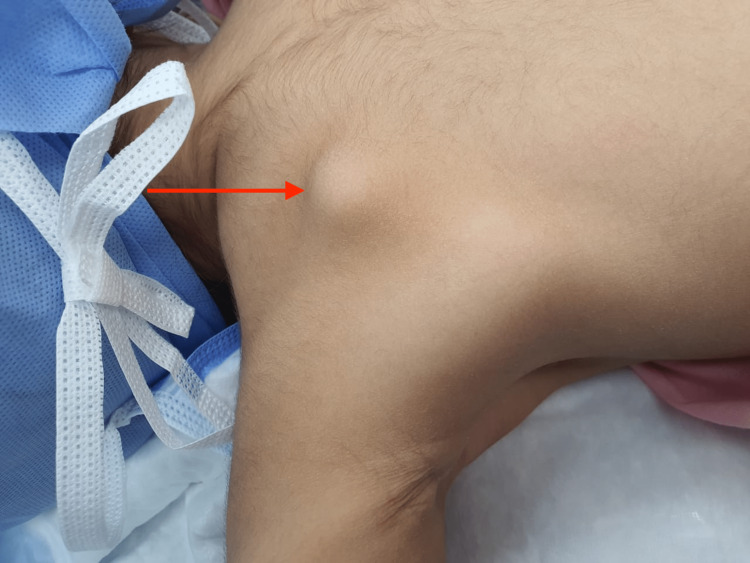
Presentation of the patient with scapular exostosis where her parents noted the lump on her back

We report a case of a two-year-old girl who presented to our clinic following her mother noticing a palpable growing and painful mass on her left scapula for 18 months. No limitation in the shoulder and scapulothoracic joint's range of motion was appreciated. No similar history among other family members was present. A well-defined round mass was seen on the dorsal aspect and palpated measuring 3 x 2.5 cm (Figure 1).

**Figure 2:**
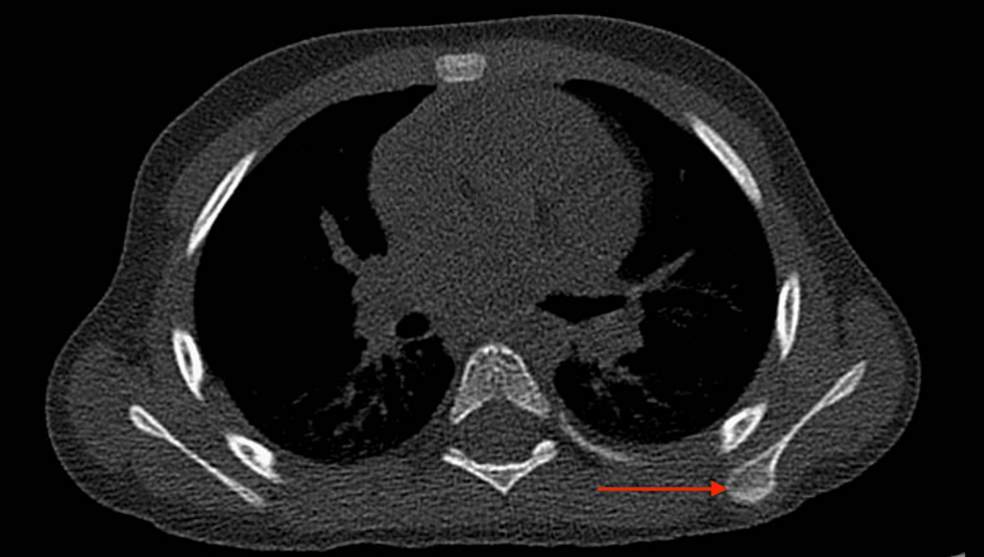
CT scan showing exostosis of the left scapula

The mass was severely painful, especially in the supine position. A CT scan confirmed the diagnosis of osteochondroma (Figure 2).

**Figure 3:**
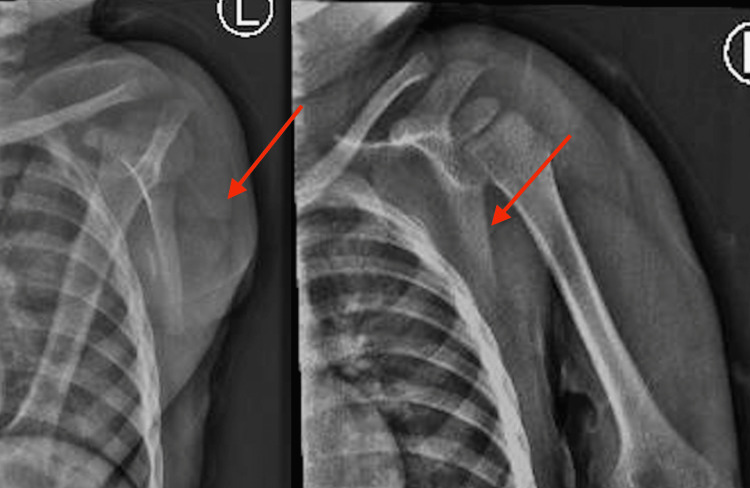
Preoperative X-ray taken for the evaluation of the patient

A nuclear bone scan was done, which concluded the presence of focal uptake in the left scapula corresponding to moderate osteoblastic activity with regular contours and mild sclerotic changes, which were likely related to exostosis, with no other lesions in the body noted on the scan. X-rays showed a bony growth on the dorsal aspect of the scapula (Figure 3).

**Figure 4:**
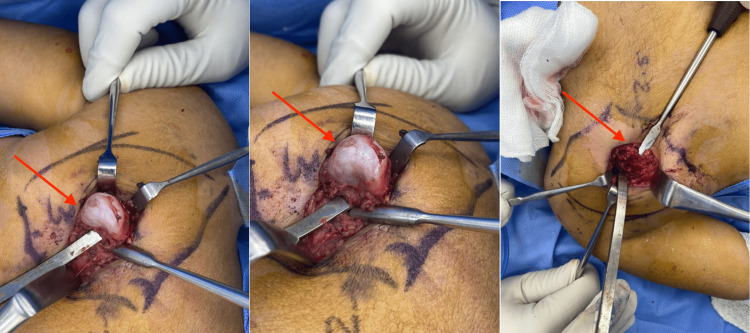
Surgical excision of the mass

The decision to go for surgical removal with a safety margin was made. The patient was placed in a prone position under general anesthesia. An incision was done right above the mass, separating the muscle directly from the mass, and excising the mass from the base so that no residual part of the mass is left behind. The stalk of the exostosis was excised at the base with an osteotome from the dorsal surface of the scapula (Figure 4). The specimen measured 3 × 2.5 cm. Histologic examination confirmed that the specimen was an osteochondroma with no signs of malignant transformation.

**Figure 5:**
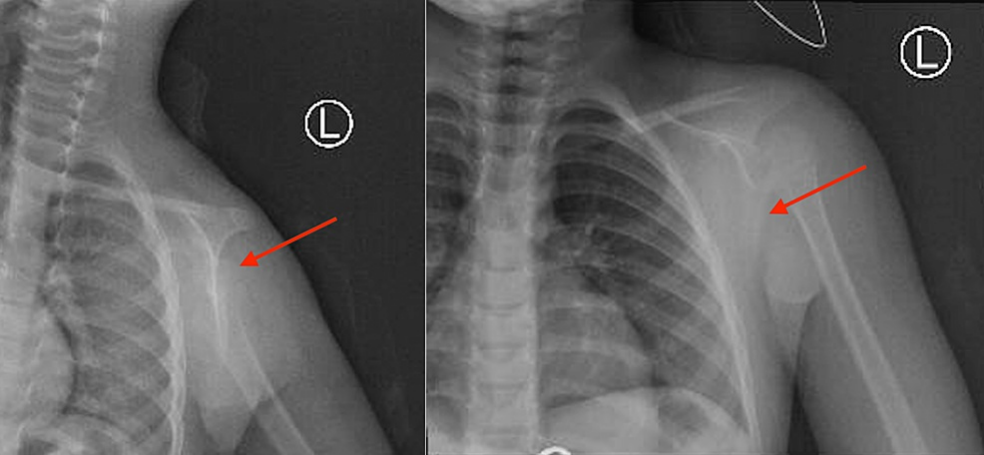
X-ray taken upon follow-up post-excision

The patient improved immediately in terms of pain and was followed up in the clinic regularly. Within almost a year, the patient had no pain, and had a full range of left shoulder motion without discomfort or pain. Follow-up X-rays showed no evidence of recurrence (Figure 5). The patient has not developed any recurrence as of now and will be continuously followed up in the clinic to check for any recurrence.

## Discussion

As noted previously, osteochondromas of the scapula account for 4% of all bone tumors occurring in the scapula [38,43]. Mostly, osteochondromas are identified in the first or second decades of life, given that the tumor’s growth usually stops when the physis closes; moreover, they are mostly asymptomatic [67,68]. Our case presents a symptomatic mass in a two-year-old noted by her mother, which in some ways is similar to multiple other papers found in the literature from different age groups, which had a similar presentation (Table 1) [14,42,43,58-61]. The novelty of our case is in the age of presentation, which is extremely rare in the literature.

**Table 1:** Literature review showing similar cases NA: not available

Author (year)	Age	Gender	Presentation	Imaging	Operative findings	Histology	Size	Follow up
Vaishya et al. (2014) [47]	18	M	Large, painless deformity, scapular winging, limited abduction of the glenohumeral joint	Medial margin	Excised specimen showing the bony tumor with a cartilaginous cap	Well-formed cartilage cap on the surface with a prominent enchondral ossification at the base, which continues into trabeculae of mature lamellar bone	5 x 3 cm	Favorable at 3 months
Ngongang et al. (2019) [48]	17	M	Worsening shoulder pain with right scapula winging	Ventromedial	The stalk of the exostosis was excised	Confirmed osteochondroma of the scapula	9 x 5 cm	Pain alleviation after two weeks, full range of motion, and better self-esteem
Sánchez et al. (2021) [50]	11	M	Painful tumor	Inferior- dorsal	After partially detaching the teres minor muscle, the tumor mass was accessed and resected en bloc	Confirmed osteochondroma of the scapula	4 x 2.8 cm	Favorable at 6 months
Bektas et al. (2019) [42]	15	F	A mass on the left upper back, inability to sleep in a supine position, painful shoulder range of motion, and cosmetic discomfort	Dorsal	Mass was excised using an osteotome	Confirmed osteochondroma of the scapula	NA	Favorable at one year
Fjeldborg et al. (2012) [51]	12	M	Winging of the scapula and minor pain	Ventral	NA	NA	NA	NA
Matthewson et al. (2019) [14]	2	M	Painless mass in his right shoulder	Dorsal	Complete resection of osteochondroma	Confirmed osteochondroma of the scapula	8.4 x 7.2 x 10.1 cm	NA
Kumar et al. (2014) [43]	4	M	Progressive swelling and movement restriction	Dorsal	Complete resection of osteochondroma	Confirmed osteochondroma of the scapula	30 x 17 mm	Favorable at 6 months
Yadkikar et al. (2013) [59]	11	F	Gradually progressive pain and inability to sleep in the supine position	Dorsal	Excisional biopsy	Confirmed osteochondroma of the scapula	3 x 2 cm	Favorable at one year
Jadhav et al. (2016) [60]	12	M	Difficulty in sleeping in the supine position	Dorsal	Excised en mass	Confirmed osteochondroma of the scapula	4 x 3 cm	Favorable at one year
Shahid et al. (2021) [58]	23	M	Painless longstanding protrusion over the scapula	Dorsal	No surgery done yet	NA	NA	NA
Nekkanti et al. (2018) [61] (Case 1)	19	M	Progressive swelling and discomfort in the supine position	Dorsal	Complete resection of osteochondroma	Confirmed osteochondroma of the scapula	3 x 3 cm	NA
Nekkanti et al. (2018) [61] (Case 2)	5	M	Progressive swelling	Dorsal	Complete resection of osteochondroma	Confirmed osteochondroma of the scapula	1.5 x 1 cm	NA

Moreover, some cases reported worsening pain (Table 1) [42,48,50,51,59], and others reported difficulty sleeping in the supine position [42,59,60]; furthermore, some cases presented limitations in the range of motion of their joints [43,47]. Most osteochondral lesions of the scapula have been noted to be situated along the scapular equator; however, larger lesions tend to be situated in the inferior aspect of the scapula due to a lack of space restriction [67,69]. Diagnosing osteochondroma is typically clinical and radiologically followed by histological confirmation [44,67,70].

Generally, osteochondromas are managed after skeletal maturity to avoid injuring the growth plate during surgery [46,71], given that with longitudinal growth, the tumor migrates from the metaphysis to the diaphysis and away from the growth plate, which decreases the chances of injuring the growth plate [71]. In the event where cosmesis and pain are the patient’s main concern in scapular exostosis, excision can be done at a younger age, such as in our case, if planned carefully and executed by the most senior surgeon of the operating team.

## Conclusions

Osteochondromas of the scapula, although benign, are at risk of being left unnoticed until malignant transformation occurs, like other central osteochondromas; therefore, we routinely advocate the removal of scapular osteochondromas at presentation. Excision after diagnosis can be done in a meticulously planned manner to avoid iatrogenic injury to the growth plate and ensure complete excision at the base of the stalk. The patient’s family is very grateful for the full recovery of their daughter and is satisfied with the results.

## References

[1] Alabdullrahman LW, Byerly DW (2022). Osteochondroma. NCBI Bookshelf [Internet].

[2] Murphey MD, Choi JJ, Kransdorf MJ, Flemming DJ, Gannon FH (2000). Imaging of osteochondroma: variants and complications with radiologic-pathologic correlation. Radiographics.

[3] Garcia RA, Inwards CY, Unni KK (2011). Benign bone tumors—recent developments. Semin Diagn Pathol.

[4] Motamedi K, Seeger LL (2011). Benign bone tumors. Radiol Clin North Am.

[5] Mirra JM (1989). Benign cartilaginous exostoses: osteochondroma and osteochondromatosis. Bone Tumors: Clinical, Radiologic, and Pathologic Correlations.

[6] Resnick D, Kyriakos M, Greenway GD (1995). Osteochondroma. Diagnosis of Bone and Joint Disorders.

[7] Milgram JW (1983). The origins of osteochondromas and enchondromas. A histopathologic study. Clin Orthop Relat Res.

[8] Keith A (1920). Studies on the anatomical changes which accompany certain growth-disorders of the human body: I. The nature of the structural alterations in the disorder known as multiple exostoses. J Anat.

[9] Hakim DN, Pelly T, Kulendran M, Caris JA (2015). Benign tumours of the bone: a review. J Bone Oncol.

[10] Murphey MD, Robbin MR, McRae GA, Flemming DJ, Temple HT, Kransdorf MJ (1997). The many faces of osteosarcoma. Radiographics.

[11] Hughes P, Dow D, Boyer L, Morganti V (2019). Ossifying chronic subperiosteal haematoma of the iliac bone. J Med Imaging Radiat Oncol.

[12] Douis H, Saifuddin A (2012). The imaging of cartilaginous bone tumours. I. Benign lesions. Skeletal Radiol.

[13] Mavrogenis AF, Papagelopoulos PJ, Soucacos PN (2008). Skeletal osteochondromas revisited. Orthopedics.

[14] Matthewson G, Singh M, Thompson S (2019). Large osteochondroma of the scapula in a 2-year-old. J Pediatr Surg Case Rep.

[15] de Souza AM, Bispo Júnior RZ (2014). Osteochondroma: ignore or investigate?. Rev Bras Ortop.

[16] Kushner BH, Roberts SS, Friedman DN (2015). Osteochondroma in long-term survivors of high-risk neuroblastoma. Cancer.

[17] Neuhauser EB, Wittenborg MH, Berman CZ, Cohen J (1952). Irradiation effects of roentgen therapy on the growing spine. Radiology.

[18] Cole AR, Darte JM (1963). Osteochondromata following irradiation in children. Pediatrics.

[19] Katzman H, Waugh T, Berdon W (1969). Skeletal changes following irradiation of childhood tumors. J Bone Joint Surg Am.

[20] Libshitz HI, Cohen MA (1982). Radiation-induced osteochondromas. Radiology.

[21] Jaffe N, Ried HL, Cohen M, McNeese MD, Sullivan MP (1983). Radiation induced osteochondroma in long-term survivors of childhood cancer. Int J Radiat Oncol Biol Phys.

[22] Paulino AC, Fowler BZ (2005). Secondary neoplasms after radiotherapy for a childhood solid tumor. Pediatr Hematol Oncol.

[23] Marcovici PA, Berdon WE, Liebling MS (2007). Osteochondromas and growth retardation secondary to externally or internally administered radiation in childhood. Pediatr Radiol.

[24] Mulcahy Levy JM, Tello T, Giller R, Wilkening G, Quinones R, Keating AK, Liu AK (2013). Late effects of total body irradiation and hematopoietic stem cell transplant in children under 3 years of age. Pediatr Blood Cancer.

[25] Shido Y, Maeda N, Kato K, Horibe K, Tsukushi S, Ishiguro N, Nishida Y (2012). Osteochondroma with metaphyseal abnormalities after total body irradiation followed by stem cell transplantation. J Pediatr Hematol Oncol.

[26] Faraci M, Bagnasco F, Corti P (2009). Osteochondroma after hematopoietic stem cell transplantation in childhood. An Italian study on behalf of the AIEOP-HSCT group. Biol Blood Marrow Transplant.

[27] Hobbie WL, Moshang T, Carlson CA (2008). Late effects in survivors of tandem peripheral blood stem cell transplant for high-risk neuroblastoma. Pediatr Blood Cancer.

[28] Trahair TN, Vowels MR, Johnston K (2007). Long-term outcomes in children with high-risk neuroblastoma treated with autologous stem cell transplantation. Bone Marrow Transplant.

[29] Flandin I, Hartmann O, Michon J (2006). Impact of TBI on late effects in children treated by megatherapy for stage IV neuroblastoma. A study of the French Society of Pediatric Oncology. Int J Radiat Oncol Biol Phys.

[30] Sanders JE, Guthrie KA, Hoffmeister PA, Woolfrey AE, Carpenter PA, Appelbaum FR (2005). Final adult height of patients who received hematopoietic cell transplantation in childhood. Blood.

[31] Taitz J, Cohn RJ, White L, Russell SJ, Vowels MR (2004). Osteochondroma after total body irradiation: an age-related complication. Pediatr Blood Cancer.

[32] Bordigoni P, Turello R, Clement L, Lascombes P, Leheup B, Galloy MA, Plenat F (2002). Osteochondroma after pediatric hematopoietic stem cell transplantation: report of eight cases. Bone Marrow Transplant.

[33] Harper GD, Dicks-Mireaux C, Leiper AD (1998). Total body irradiation-induced osteochondromata. J Pediatr Orthop.

[34] Maeda G, Yokoyama R, Ohtomo K, Takayama J, Beppu Y, Fukuma H, Ohira M (1996). Osteochondroma after total body irradiation in bone marrow transplant recipients: report of two cases. Jpn J Clin Oncol.

[35] Fletcher BD, Crom DB, Krance RA, Kun LE (1994). Radiation-induced bone abnormalities after bone marrow transplantation for childhood leukemia. Radiology.

[36] Tittal P, Pawar I, Kapoor SK (2015). Pseudo-winging of scapula due to benign lesions of ventral surface of scapula - two unusual causes. J Clin Orthop Trauma.

[37] Calafiore G, Calafiore G, Bertone C, Urgelli S, Rivera F, Maniscalco P (2001). Osteochondroma. Report of a case with atypical localization and symptomatology. (Article in Italian). Acta Biomed Ateneo Parmense.

[38] Galate JF, Blue JM, Gaines RW (1996). Osteochondroma of the scapula. Mo Med.

[39] Rameez R, Ul-Hassan M, Kotwal HA, Kangoo KAH, Nazir A (2016). Painful pseudowinging and snapping of scapula due to subscapular osteochondroma: a case report. J Orthop Case Rep.

[40] Tomo H, Ito Y, Aono M, Takaoka K (2005). Chest wall deformity associated with osteochondroma of the scapula: a case report and review of the literature. J Shoulder Elbow Surg.

[41] Sivananda P, Rao BK, Kumar PV, Ram GS (2014). Osteochondroma of the ventral scapula causing scapular static winging and secondary rib erosion. J Clin Diagn Res.

[42] Bektas YE, Ozmanevra R (2019). An unusual location of osteochondroma: dorsal scapula. Cureus.

[43] Kumar C Y, Shervegar S, Gadi D, Rahul P (2014). Solitary sessile osteochondroma of scapula, a rare case report. J Clin Diagn Res.

[44] Kitsoulis P, Galani V, Stefanaki K, Paraskevas G, Karatzias G, Agnantis NJ, Bai M (2008). Osteochondromas: review of the clinical, radiological and pathological features. In Vivo.

[45] Tepelenis K, Papathanakos G, Kitsouli A (2021). Osteochondromas: an updated review of epidemiology, pathogenesis, clinical presentation, radiological features and treatment options. In Vivo.

[46] Khare GN (2011). An analysis of indications for surgical excision and complications in 116 consecutive cases of osteochondroma. Musculoskelet Surg.

[47] Vaishya R, Dhakal S, Vaish A (2014). A solitary osteochondroma of the scapula. BMJ Case Rep.

[48] Ngongang FO, Fodjeu G, Fon AC, Fonkoue L, Guifo ML, Bitang A Mafok LJ, Ibrahima F (2019). Surgical treatment of rare case of scapula osteochondroma in a resource limited setting: A case report. Int J Surg Case Rep.

[49] Okada K, Terada K, Sashi R, Hoshi N (1999). Large bursa formation associated with osteochondroma of the scapula: a case report and review of the literature. Jpn J Clin Oncol.

[50] Segura Sánchez D, Pino Almero L, Mínguez Rey MF (2021). A solitary osteochondroma of the scapula: uncommon location for a common tumor. Arch Argent Pediatr.

[51] Fjeldborg PK, Hansen TB (2012). Atypical cause of scapular winging due to exostosis of the scapula. (Article in Danish). Ugeskr Laeger.

[52] Alrashedan BS, Chowdhary SK, Mahmoud J, Hamza OM (2019). Symptomatic osteochondroma in the ventral aspect of the scapula in a child with hereditary multiple exostoses. J Musculoskelet Surg Res.

[53] El Rharras S, Farah R, El Haoury H, Saidi H, El Bouchti I (2017). Solitary osteochondroma of the scapula: an uncommon localization. Clin Case Rep Rev.

[54] Pérez D, Cano JR, Caballero J, López L (2011). Minimally-invasive resection of a scapular osteochondroma. Interact Cardiovasc Thorac Surg.

[55] Dharmadhikari RP (2012). Painful snapping and pseudo-winging scapula due to a large scapular osteochondroma. J Orthop Case Rep.

[56] Ermiş MN, Aykut US, Durakbaşa MO, Ozel MS, Bozkuş FS, Karakaş ES (2012). Snapping scapula syndrome caused by subscapular osteochondroma. Eklem Hastalik Cerrahisi.

[57] Chillemi C, Franceschini V, Ippolito G, Pasquali R, Diotallevi R, Petrozza V, Rocca CD (2013). Osteochondroma as a cause of scapular winging in an adolescent: a case report and review of the literature. J Med Case Rep.

[58] Shahid O, Shahid M, Shaik L, Masud M, Ranjha S (2021). Rare case of osteochondroma on the dorsal aspect of the scapula. Cureus.

[59] Yadkikar SV, Yadkikar VS (2013). Osteochondroma on dorsal surface of the scapula in 11 years old child-a case report. Int J Med Res Heal Sci.

[60] Jadhav PU, Banshelkikar SN, Seth BA, Goregaonkar AB (2016). Osteochondromas at unusual sites- case series with review of literature. J Orthop Case Rep.

[61] Nekkanti S, Moogali A, Meka A, Nair M (2018). An unusual presentation of osteochondroma on the dorsal surface of the scapula: a review of two patients. J Orthop Case Rep.

[62] Danielsson LG, El-Haddad I (1989). Winged scapula due to osteochondroma. Report of 3 children. Acta Orthop Scand.

[63] Bovée JV (2008). Multiple osteochondromas. Orphanet J Rare Dis.

[64] Lotfinia I, Vahedi P, Tubbs RS, Ghavame M, Meshkini A (2010). Neurological manifestations, imaging characteristics, and surgical outcome of intraspinal osteochondroma. J Neurosurg Spine.

[65] D'Arienzo A, Andreani L, Sacchetti F, Colangeli S, Capanna R (2019). Hereditary multiple exostoses: current insights. Orthop Res Rev.

[66] Wolford LM, Movahed R, Dhameja A, Allen WR (2014). Low condylectomy and orthognathic surgery to treat mandibular condylar osteochondroma: a retrospective review of 37 cases. J Oral Maxillofac Surg.

[67] Alatassi R, Koaban S, Almugebel I, Alshehri A (2018). Scapular osteochondroma with winging: a case report. Int J Surg Case Rep.

[68] Fletcher CD, Unni KK, Mertens F (2002). Pathology and Genetics of Tumours of Soft Tissue and Bone. https://www.google.co.in/books/edition/Pathology_and_Genetics_of_Tumours_of_Sof/dg9am0g4EP8C?hl=en&gbpv=0.

[69] Nascimento AT, Claudio GK (2017). Snapping scapula. Arthroscopic resection of osteochondroma of the subscapularis superomedial angle. Case report and literature review. Rev Bras Ortop.

[70] Kwon OS, Kelly JI (2012). Delayed presentation of osteochondroma on the ventral surface of the scapula. Int J Shoulder Surg.

[71] Natrajan MV (1994). Natarajan’s Text Book of Orthopaedics and Traumatology. Natarajan’s Text Book of Orthopaedics and Traumatology.

